# Influence of Fabric Support on Improving the Layer-by-Layer Polyethersulfone Membrane Performance

**DOI:** 10.3390/polym17212825

**Published:** 2025-10-23

**Authors:** Ahmed A. Bhran, Abdelrahman G. Gadallah, Eman S. Mansor, Heba Abdallah

**Affiliations:** 1 Chemical Engineering Department, College of Engineering, Imam Mohammad Ibn Saud Islamic University (IMSIU), Riyadh 11432, Saudi Arabia; agadallah@imamu.edu.sa; 2 Water Pollution Research Department, Environment and Climate Change Research Institute, National Research Centre, Giza 12622, Egypt; eman_mansor31@yahoo.com; 3 Chemical Engineering & Pilot Plant Department, Engineering Research Institute, New and Renewable Energy, National Research Centre, Giza 12622, Egypt; heba_nasr94@yahoo.com

**Keywords:** fabric support, woven, non-woven, membrane, polyester, polypropylene

## Abstract

This work is based on studying the effect of different kinds of support on the prepared reverse osmosis membranes. Different kinds of woven and non-woven supports were tested and characterized to select the best one for RO membrane preparation. The prepared membrane on polyester woven support (M1ws) provides 39.9 LMH permeate flux using a piperazine coagulation bath during membrane preparation, while polyester non-woven support (M2ns) exhibits the highest salt rejection percentage, which was 92.2% using a Melamine coagulation bath. The mechanical properties for preparing membranes using supports were arranged in descending order as follows: M1ws > M2ns > M3np. The membrane on polypropylene support (M3np) provides the lowest mechanical properties.

## 1. Introduction

Human existence is based on clean water as a vital raw material for different businesses, electronics, medicine, and food, among others [1].

Numerous water-related issues are plaguing the world, such as the declining need for pure water due to prolonged scarcities, growth in population, more stringent health-based regulations, and competing requests from several users. Seawater is the most readily available form of water, while freshwater makes up only 3% of the planet’s total water supply [2]. Thus, the most interesting and promising technological advancements are those utilized in water desalination [1,2]. Membrane technology’s low thermal energy consumption and excellent water treatment purity make it a potential solution to replace the current conventional treatment methods [3].

The osmosis phenomenon is used in the physical process known as reverse osmosis (RO), which eliminates salts from water by reversing the flow of pure water at a pressure greater than the osmotic pressure, letting freshwater flow through the synthetic membrane’s pores while keeping the salt out. Both brackish and seawater can be desalinated using the RO method [4,5].

Recently, the use of supporting materials to enhance membrane characterization was studied. For example, The dispersion of nanofibers in a solution was studied using a high-speed shear machine to obtain a 2 wt% nanofiber suspension; this dispersion was sprayed in wet form onto a polyethylene terephthalate non-woven fabric surface. After that, the interfacial polymerization process was carried out using piperazine and 1,3,5-benzoyl chloride, where this kind of membrane was used for Congo Red dye separation of 98.9%. In this case, the produced membrane has a porous structure and cannot be used in salt separation because of the low rejection of NaCl of 6.5% [6]. MXene layer was prepared using Ti_3_AlC_2_ powder which was added into the Polytetrafluoroethylene (PTFE) solution. This layer was added on the polyether sulfone membrane surface to enhance the surface. The thickness of the film layer was adjusted by controlling the amount of MXene deposited onto polyether sulfone (PES) membrane surface by vacuum filtration. This kind of composite membrane delivered a 99% crystal violet dye rejection rate and a porous surface [7]. The addition of MXene nanosheets altered the membrane’s characteristics and improved its physicochemical properties. The modified membrane’s water flux was increased by almost 80% while its reverse solute flux was marginally higher than that of the unmodified TFC-FO membrane. Additionally, its particular reverse osmosis flux (only 0.63 g/L) was greatly optimized [8]. Biopolymer support preparation was studied using chitosan and cellulose to produce porous membrane for interfacial polymerization process. The chitosan membrane was used to support a thin-film composite layer produced by the means of interfacial polymerization, reaching a high rejection rate of 99.4% of NaCl (2000 ppm). Nevertheless, this membrane with no fabric support has a shorter life and exhibits unstable mechanical characteristics [9].

Both woven and non-woven fabrics can be utilized as support for the preparation of polymeric membranes. Non-woven fabrics are composed primarily or exclusively of main fibers, such as small fleece fibers that undergo treatment with particular fiber types, provided that this treatment has no appreciable impact on the fleece’s capacity to consolidate [10]. Two common kinds of non-woven fabrics are prepared from polyester or polypropylene fibers, which belong to the group of polyolefins and are partially crystalline and non-polar. Polypropylene (PP) fibers are slightly harder, more heat-resistant, and mechanically rugged materials and have a high chemical resistance [11]. Polypropylene has excellent isolative properties due to its hydrophobic nature. Although polypropylene lacks a real softening point temperature, its melting point is roughly 165 °C, and the fiber’s maximum processing temperature is 140 °C. However, after submerging PP fibers in water for 24 h, they absorb about 0.3% of the water; therefore, it is considered a hydrophobic polymer. Polypropylene fibers are unaffected by water. Most acids, alkalis, and salts do not affect polypropylene fiber, which also exhibits exceptional resilience to most acids except for strong sulfuric acid and chlorosulfonic acid [12,13,14]. A non-woven fabric’s fibers can be arranged randomly or in a single direction.

To provide the required strength, elongation, and other mechanical characteristics, several layers might be joined. Fiber diameter, density, orientation, and extra mechanical processing can all be used to modulate porosity [11,12,13,14]. Although woven fabrics are the final products of spinning and weaving, they are also used as raw materials in the apparel industry as well as other sectors like composites and medical textiles. Polyester woven fabric, one of the various varieties of woven fabrics, is commonly utilized as a substrate for polymer coatings [11,12,13,14,15,16]. The repetition unit of polyester is rather straightforward [17]. Polyester woven fabrics have a static electrostatic charge on their surface, which means that, because they are hydrophobic, they retain more static charge and have poor moisture absorption capabilities. When woven fabrics come into contact with other materials, such as polymers, they either have a tendency to capture electrons and become negatively charged (−) or to provide electrons and become positively charged (+) [18]. On the other hand, when polyester woven fabrics come into contact with other materials, they attract electrons and develop negative electrical charges.

To balance the electrochemical potential, electrical charges go from the polymeric solution to the fabric when the polymeric solution and fabric support come into contact [18,19,20,21,22]. The main obstacle to osmotic flow is the porous substructure of membranes. The membranes’ functioning was always restricted in the absence of the woven or non-woven porous support fabric that is uniformly applied to all commercially available membranes by attaching to the structural side.

This paper is devoted to polyethylene sulfone (PES) membrane preparation with the help of textile materials of different structures, thicknesses, and types, such as woven and non-woven fabric. The addition of TiO_2_ and polyethyleneimine (PEI) as additives to the polymer-based blend is a new strategy to achieve adjustable surface charge by adjusting the hydrophilicity and mechanical properties of the membrane before coating. PEI also gives a positive charge to the membrane surface; however, in combination with TiO_2_, the surface charge is now adjustable, depending on the pH of the feed solution, allowing the separation of different salts effectively. Also, the incorporation of melamine or piperazine in the coagulation step prior to coating favors the penetration of amine monomers into the base membrane in the solvent/non-solvent exchange process, producing a stable, smooth surface with highly organized pores [23,24]. The impact of employing fabric support during the manufacturing of reverse osmosis membranes was studied based on the mechanical characteristics and improved membrane performance. Characterization of membranes and supports was carried out. An examination of various coagulation baths was conducted.

## 2. Materials and Methods

### 2.1. Materials

PES Ultrason 6020 (polyethersulfone) was acquired from BASF company (Frankfurt, Germany). The solvent, N-Methyl Pyrrolidone (NMP), was obtained from Sigma Aldrich Company, Darmstadt, Germany. Fluka Company (Neu-Ulm, Germany) was the supplier of sodium dodecylsulfate (SDS), polyethyleneimine, and trimesoyl chloride. Sigma Aldrich Company, (Darmstadt, Germany) was the supplier of melamine, piperazine, cyclohexane, sodium hydroxide, and hydrochloric acid.

### 2.2. Characterization

SEM was utilized to show the structure of the membranes and fabric support. With the use of QUANTA FEG250 (San Francisco, CA, USA) scanning electron microscope delivered from Thermo Fisher Scientific company, the samples were coated with gold to create electrical conductivity.

Contact angle measurement was used to indicate the hydrophilic feature and wetting ability of the support surface. A compact video microscope (CVM) made in Libertyville, IL, USA by the Penco Precision company was used to determine the contact angle. The contact time was 10 s with an average drop volume of 10 μL, and each value was averaged from 10 repeated measurements.

The mechanical characteristics of various fabric supports and the produced membranes were examined. A Tinus Olsen company mechanical testing system model H5KS made in Birmingham, AL, USA was used to determine the supports’ tensile strength and elongation. The average values of the four samples for each support were calculated with a standard error deviation of ±5.

The fabric support surface was subjected to FTIR examination using a Bruker VERTEX 70 FTIR spectrometer (Bruker Optics GmbH, Ettlingen, Germany) with a resolution of 4 cm^−1^ and a scanning rate of 16 scans/min.

### 2.3. Support Permeability Test and Water Uptake %

**Figure 1:**
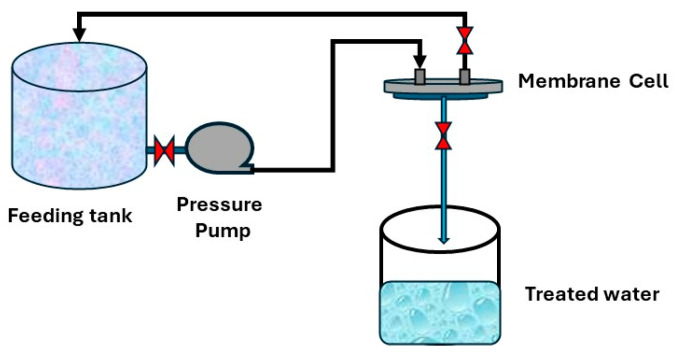
Permeability testing system.

Distilled water was used to evaluate the samples for permeability. Four samples for each support were tested, and the average of readings was recorded. A filtration lab unit with a 5 cm diameter was used for the test. There are three holes in this unit: one for feeding, one for rejection, and one for permeate, as shown in Figure 1. Equation (1) was used to calculate the flux:

(1) $$J=\frac{V}{\left(A\times T\right)}$$

where J is the permeate flux (L/m^2^.h), q is the product volume, A is the active surface area of the membrane (m^2^), and T is time (h).

Following a 24 h soak in water, the samples’ degree of water uptake was assessed, and any surface solution was swiftly and carefully removed from the swollen samples using tissue paper. A digital analytical balance was then used to weigh the wet fabric. The wet fabrics were then dried entirely for 24 h at 80 °C in an air-circulating oven, and the dried samples were weighed. Equation (2) was used for WC calculation:

(2) $$WC\%=\frac{\left[W_{wet}-W_{dry}\right]*100}{W_{wet}}$$

where WC is the water uptake degree and Wwet and Wdry are the fabric’s dry weight (g) and wet weight (g). The support porosity (ε) was determined by dividing the difference between the wet and dry weights of the fabrics with the sample area (A) and thickness (x), as illustrated in Equation (3) [25].

(3) $$\varepsilon \left(\%\right)=\frac{Wwet-Wdry}{\;A\times x}\times 100$$

### 2.4. Membrane Preparation

Phase inversion was used to create reverse osmosis membranes. The polymer solution was made by dissolving 1 weight percent polyethyleneimine (PEI) and 0.3 wt% sodium dodecyl sulfate with 0.5 weight percent TiO_2_ in NMP solvent while being sonicated for 20 min. After adding 24 wt% of polyethersulfone to the polymeric mixture, it was agitated for eight hours with a mechanical stirrer. In the casting process, three types of supports were used: polyester woven, polyester non-woven, and polypropylene non-woven. A casting knife was used to cast the support, which was mounted on a glass plate and had a thickness of 200 µm.

**Figure 2:**
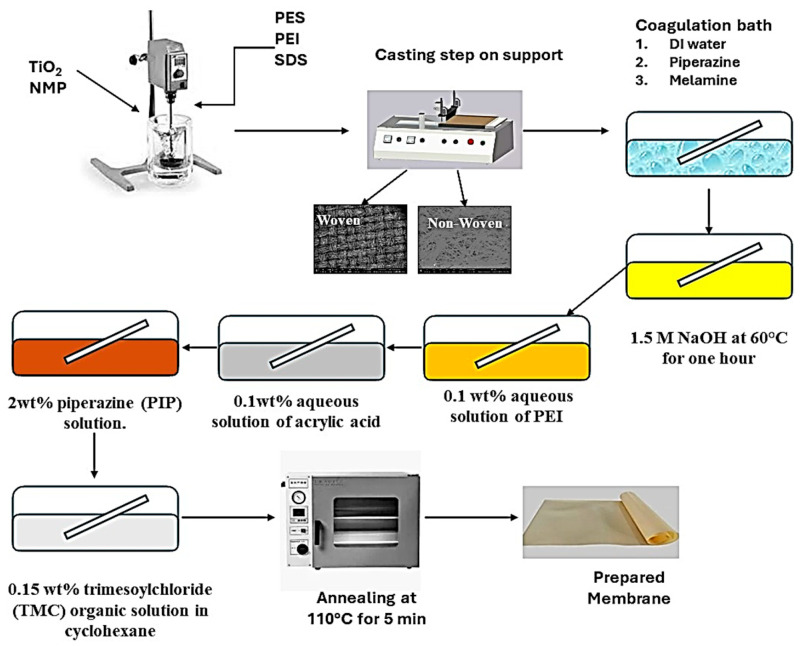
Membrane preparation steps.

Distilled water baths containing 0.1 wt% piperazine or 0.1 wt% melamine were used to investigate the coagulation process. After 10 min of coagulation, for later use, the membrane was stored in distilled water for a full day. To finish the layer-by-layer coating process, the produced membranes were immersed in 1.5 M NaOH at 60 °C for an hour. The membranes were then immersed for 15 min at 30 °C in a 0.1 weight percent aqueous solution of PEI at pH 12.6. The membranes were then allowed to soak for 20 min at 30 °C in a 0.1 wt% aqueous solution of acrylic acid. Finally, interfacial polymerization was performed on the membrane surface using 0.15 weight percent trimesoyl chloride (TMC) organic solution in cyclohexane and 2 wt% piperazine (PIP) solution to finish the coating process. The annealing step of the membrane was performed at 110 °C for 5 min. Table 1 illustrates the prepared membranes using different supports, and Figure 2 illustrates the membrane preparation steps.

### 2.5. Membrane Performance Test

**Figure 3:**
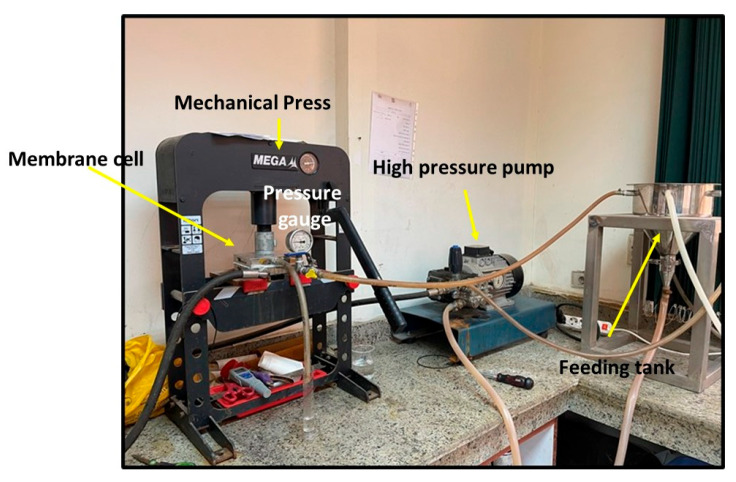
Setup of the desalination unit.

The performance test of the membranes was conducted using synthetic salt solution of 5000 ppm NaCl. Figure 3 depicts the National Research Center’s laboratory desalination testing facility in Egypt. It features three feeding, concentrate, and permeate apertures in a flat sheet membrane module with an area of 89.9 cm^2^. The feed solution tank has a capacity of 10 L, and the performance tests were conducted at room temperature with an operating pressure of up to 25 bar. Equation (1) was used to calculate the tested salty solution’s total flux (J), while Equation (4) was used to compute the rejection (R).

(4) $$R\%=\frac{\left(C_{f}-C_{p}\right)*100}{C_{f}}\;$$

where *C_f_* is the feed concentration and *C_p_* is the product concentration.

Long-term experiments were carried out on the three prepared membranes in a melamine bath using different fabric supports. The experiments were conducted at 25 bars with a 5000 ppm NaCl synthetic solution over a 5 h operation period.

## 3. Results and Discussions

### 3.1. Support Characterization

#### 3.1.1. Scanning Electron Microscope and Contact Angle for Supports

Table 2 illustrates the surface morphology and the thickness of fibers for different types of fabric (woven and non-woven). The woven fabric appears less compatible than the non-woven, and its fiber thickness is greater than the commercial non-woven one.

Non-woven fabrics exhibit more compacted fibers than woven fabrics. However, Polypropylene support has larger non-woven fibers with an average thickness of 25.9 µm in comparison with polyester non-woven fabric, which is 8.58 µm. The difference in the fiber thickness is related to the textile fabrication process. To enable the casting of a polymeric solution on this support, the membrane support fabrics need to be highly consistent and have a flawless surface. The surface needs to be incredibly smooth and flat, free of any loose or standing fibers. The largest persistent issue facing membrane makers is standing fibers.

During the casting process, the polymer cannot form an uninterrupted surface free of imperfections if individual fibers or groups of fibers are loose or protrude above the plane of the substrate. These surface flaws usually result in a defective module by causing pinholes or bigger cavities in the membrane, in addition to a loss of surface integrity at the location of imperfection [10,17]. The contact angle of the supports was investigated, where the polyester non-woven exhibits 89.6°, and polypropylene 93.4°, while the woven support exhibits a contact angle of 84.5°. The contact angle exhibits a hydrophobic nature, which can be considered suitable for a coating layer using a polymeric solution.

Accordingly, the support used in the membrane fabrication should mostly have more compacted fibers, such as non-woven support with low fiber thickness to create uniformity for the membrane surface by reducing the spaces between fibers and creating a smooth surface to cast the polymeric solution over the support surface or stabilize the coating. However, using woven support has a smooth surface, which can also lead to high permeate flux due to the woven structure, which provides good organization of the fabric distribution [17,18].

#### 3.1.2. Fabric Support Mechanical Properties

**Figure 4:**
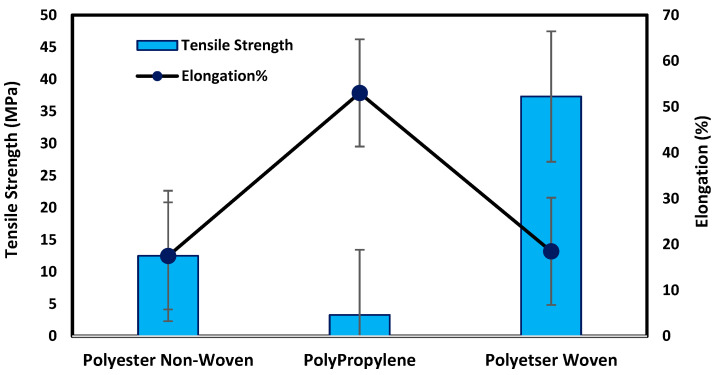
Mechanical testing for fabric supports.

Figure 4 illustrates that the woven support exhibits the highest mechanical properties compared with non-woven supports due to the fabric spinning, as shown in SEM. The tensile strength and elongation percentage of each support can be arranged as follows: polyester woven > polyester non-woven > polypropylene non-woven. Polyester woven exhibits 37.3 MPa and elongation of 18.5% while the non-woven polyester provides 12.5 MPa and 17.5% for tensile strength and elongation percentage, respectively. The results indicate that the woven polyester exhibits resistance to shrinking, stretching, mildew, and creasing. Stretching properties lead to an improvement in the mechanical properties of the fabric; hence, it can be applied to the fabrication of membranes, particularly those that need high pressure to function. However, non-woven fabrics have the merits of dimensional stability, strength, durability, resilience, and porosity level controlled based on specific processing. Accordingly, most non-woven support can be used in membrane fabrication, especially for membranes that require low porosity to improve the dense or selective layer of membranes [14,15,16,17,18].

#### 3.1.3. Fourier Transform Infrared (FTIR) Spectrophotometer

**Figure 5:**
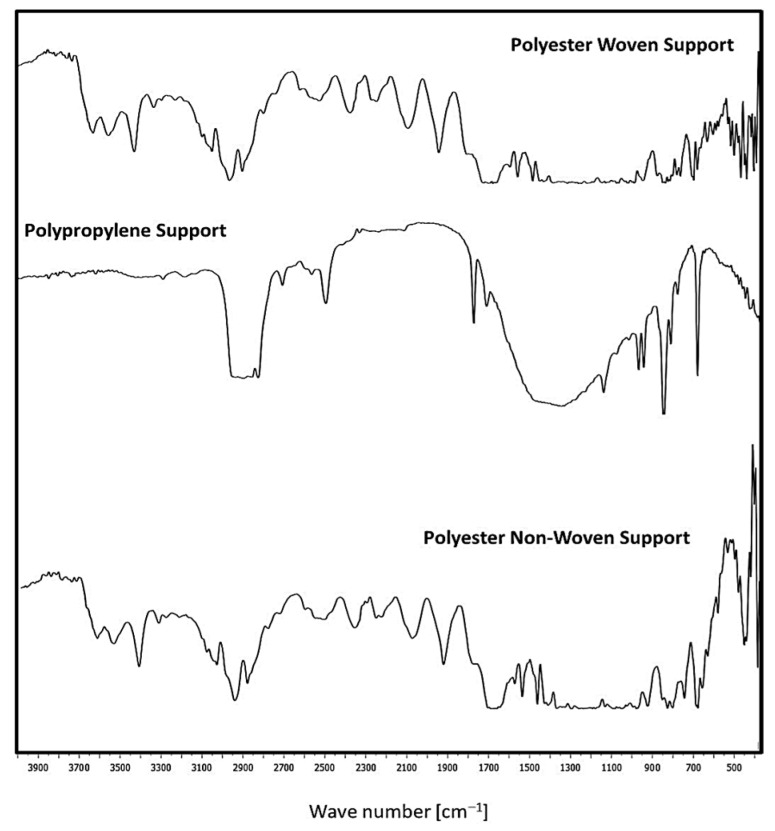
FTIR for the different fabric supports.

Figure 5 illustrates the FTIR analysis for fabric supports. Woven and non-woven polyester supports exhibit peak stretching chains from 726 cm^−1^ to 1696 cm^−1^ due to the presence of the C=O group, C=C stretching, and C=C bending, respectively. Also, this support is an aliphatic polyester since it has two peaks at 2969 cm^−1^ and 846 cm^−1^, which show the existence of a CH_2_ group. Also, it was noticed that the para-substituted benzene is indicated by the appearance of two peaks at 1570 cm^−1^ and 1504 cm^−1^ [5,26,27,28].

On the other hand, the C=N stretch vibration group is represented by the band at 1577 cm^−1^. Nonetheless, the aromatics and alkanes of ethylene glycol (OH-CH_2_-CH_2_-OH) are responsible for the presence of peaks at 1470 cm^−1^ and 1411 cm^−1^. The presence of the aldehyde, C≡N, C=N, and O-CH groups is shown by the peaks at 2700–2800 cm^−1^ and 2389 cm^−1^. The existence of OH, NH, and CH groups is shown by the peaks in the 3000–3500 cm^−1^ range. Furthermore, the presence of the peaks at 2283–3398 cm^−1^ is consistent with C=N [5]. Polypropylene support consists of peaks characteristic for CH_2_- group (2916 cm^−1^, 2841 cm^−1^), >CH- group (1170 cm^−1^, 975 cm^−1^, 899 cm^−1^, 841 cm^−1^, 810 cm^−1^) and –CH3 group (2959 cm^−1^, 2881 cm^−1^, 1560 cm^−1^, 1476 cm^−1^) [29].

#### 3.1.4. Permeability Test and Water Uptake %

**Figure 6:**
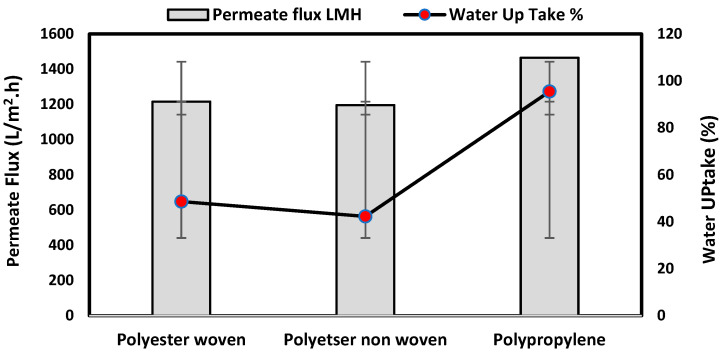
Permeability test and water uptake% for fabric support.

The water uptake percentage and permeate flow of various fabric supports are illustrated in Figure 6, and the results can be arranged as follows: polypropylene non-woven > polyester woven > polyester non-woven. These results agree with the SEM, where the fiber thickness of the polypropylene non-woven (PP) sample was the highest (25.92 µm) and the fiber thickness of the polyester non-woven was the lowest, showing a value of 8.58 µm. From the SEM, the non-woven polyester support is very compacted, while the sample of polypropylene was loose with thick fibers. Hydrophobic support can provide a good chemical bond between the support and the polymeric casting layer. The lower water uptake percentage means the higher the hydrophobic properties of the fabric support surface.

These rounded and smooth fibers of woven polyester reduce drag resistance, which increases the spreading and transportation of liquids. They are also high in mechanical strength and porosity, and strong in supporting membranes and other applications, which enhances flux.

Table 2 also indicates the contact angle for the fabric support, which indicates the PP sample has the highest hydrophobicity, which means this sample can accept the polymeric layer of the membrane; however, due to the highest thickness of the fabric and low compacting between fabrics, the permeate flux of water was higher than other samples.

The materials of the support during membrane casting are mostly completely covered by the polymeric solution based on the mass of the casting solution, the wet thickness of the polymer film, and the working environment, so all open mesh fabrics may be used as membrane support (woven or non-woven) based on the nature of materials and their compatibility together [30,31,32]. The porosity of the supports was determined as follows: non-woven polyester support 82.2%, woven polyester support 89.5% and non-woven polypropylene support 90.7%. Higher fiber diameter is proportional to larger fabric porosity, which was seen in the non-woven polypropylene.

### 3.2. Prepared Membranes

#### 3.2.1. Scan Electron Microscope Analysis for Prepared Membranes

**Figure 7:**
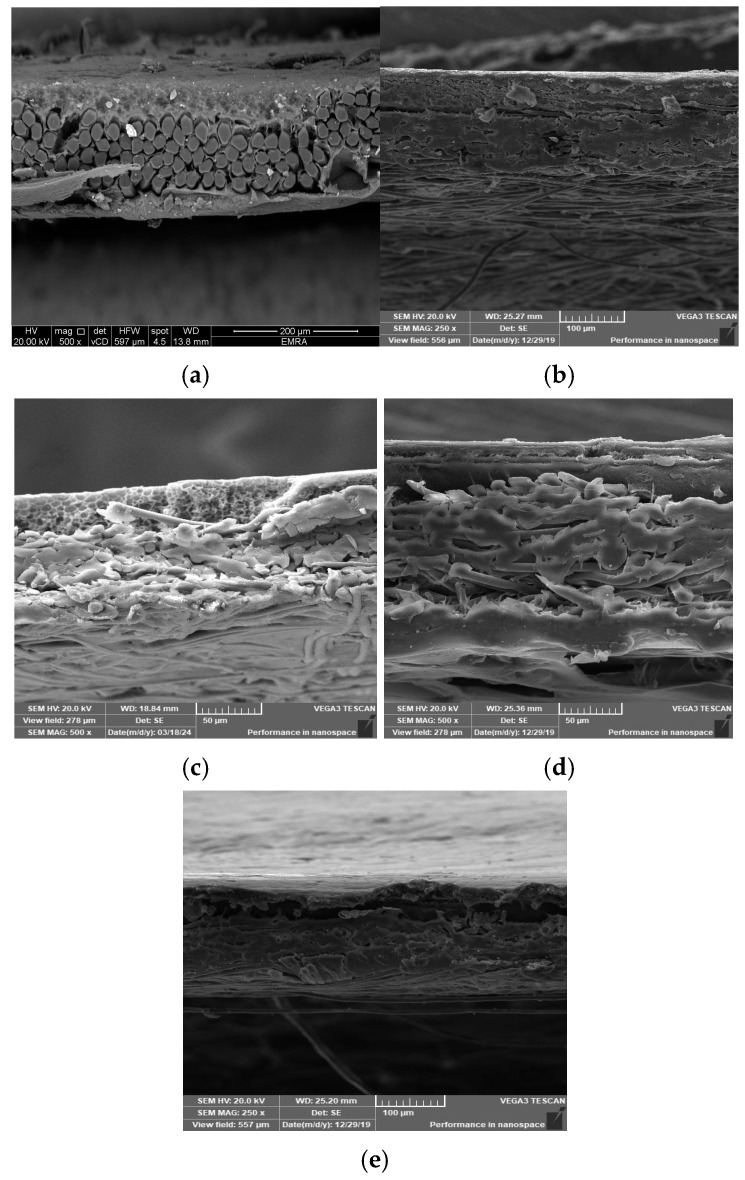
Cross-section SEM images of the prepared membrane: (**a**) using polyester woven support, (**b**) polyester non-woven support, (**c**) using polypropylene support, (**d**) using melamine bath, and (**e**) using piperazine bath.

Five membrane cross-sections were snapped up to indicate the inner structure of membranes. A cross-section of the constructed membrane utilizing a woven polyester support is shown in Figure 7a, where the membrane’s top layer is dense, its sub-layer structure is spongy, and the woven support’s fibers are visible in the bottom layer. Figure 7b indicates that the same membrane was cast on the polyester non-woven support, where the well-compacted fibers appear at the bottom of the membrane. Figure 7c illustrates that the membrane was cast on the polypropylene support, where the compacted thick fibers appear on the bottom. Fibers that intermittently or vertically protrude upward from the horizontal fabric coating surface plane are often problematic because, until they are flattened into the web by flame-singeing, post-calendaring, or another technique, these fibers permit the polymeric solution to move away from the fiber or flow through it. During the casting process, pinholes and other flaws appear as the polymeric solution starts to solidify [17,18]. Figure 7d,e exhibit a highly dense top layer due to the use of a layer-by-layer coating process, where using piperazine in the coagulation bath exhibits a thicker top layer than using a melamine bath.

#### 3.2.2. Mechanical Testing for Prepared Membranes

**Figure 8:**
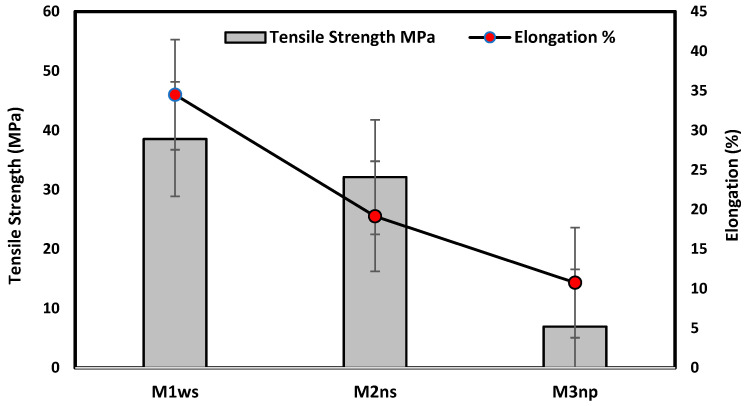
Mechanical testing for prepared membranes.

Figure 8 illustrates that the prepared membrane on the woven support exhibits the best mechanical properties, with a tensile strength of 38.53 MPa and an elongation of 34.5%. This type of support enhances the elasticity of the membrane, as shown in Figure 4, due to the nature of the fibers on the support. The woven support also exhibits the highest mechanical properties. However, the membranes cast on the non-woven polypropylene support had the lowest mechanical qualities due to the high thickness of fibers and being less compacted in comparison with the polyester non-woven support, as shown in Figure 4 and Figure 8. According to membranes related to support, the mechanical properties can be set up in the following manner: M1ws > M2ns > M3np. The smooth and well-organized structure of the woven fabric increased the elasticity of the membranes prepared on them in comparison to compressed non-woven fabrics that have disorganized fibers and may decrease membrane elasticity [17].

#### 3.2.3. Membrane Performance Test

**Figure 9:**
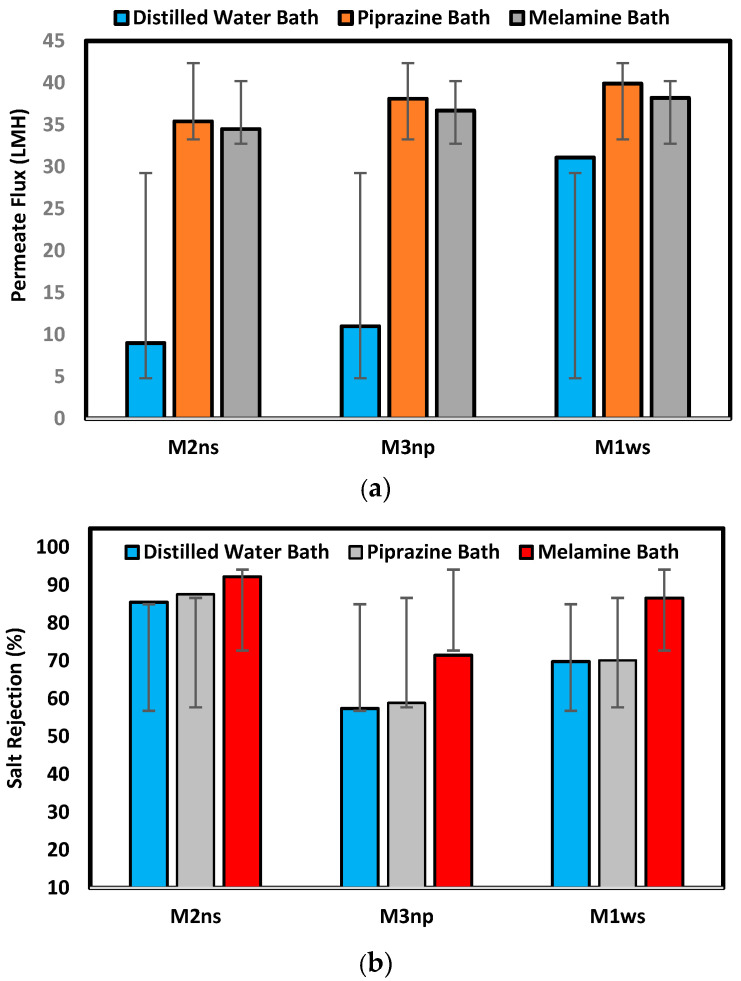
Membrane performance test in terms of (**a**) permeate flux (L/m^2^.h) and (**b**) salt rejection percentage.

Figure 9 illustrates the membrane performance testing on the prepared membranes with different supports and coagulation baths. The findings show that, among all produced membranes on woven support, M1ws offers the best permeate flux, which agrees with the characterization of this kind of support, where the permeate flux reached 31.1, 39.9, and 38.2 L/m^2^.h for prepared membranes in different baths of water, piperazine, and melamine, respectively. The membranes prepared in a distilled water coagulation bath had a permeate flux of 31.1 L/m^2^.h, 11 L/m^2^.h and 9 L/m^2^.h for M1ws, M3np and M2ns, respectively. The highest permeate flux was observed on the woven-supported membrane (M1ws) because there was less resistance to mass transfer, which increases the permeability. The woven fabric allows the steady flow of fluids through the capillary openings of the fabric [33,34].

On the other hand, M2ns exhibits the highest salt rejection percentage, which was 85.5%, 87.6%, and 92.2% for the prepared membranes in different baths: water, piperazine, and melamine baths, respectively. During membrane creation, the non-woven polyester support’s compacted structure stops the polymeric solution from penetrating through it, as shown in the SEM images in Figure 6. Three layers make up the membrane structure, a dense top layer, a sublayer, and a porous bottom support layer, with each layer doing their work. However, the layer-by-layer coating provides a highly dense selective layer which leads to high salt rejection [17,18]. Using the woven polyester support (WPS), the polymeric solution enters the interstitial spaces between the yarns of the fabric, which introduces defects in the membrane layers, lowering the efficiency in the salt rejection. M3np, which represents a membrane supported by polypropylene, provides low flux and rejection percentage due to its less compacted structure and the high thickness of the compacted fibers [5,27,28,29].

Any variation in the web’s thickness from side to side makes it difficult to cast the polymeric membranes, as it can result in thin fabric sections where the polymer puddles in low places or valleys. High spots throughout the width cause a thin polymer covering and the possibility of pinholes. It becomes difficult to roll the coated membrane into a spiral with consistent stiffness and tension, or nearly impossible when the fabric thickness varies across its width. Fabric flaws are serious quality issues that, if left unaddressed, are the primary reason for membrane rejection and/or eventual field failure. Frequently, defects are undetected until a module is put through initial or follow-up cleaning procedures [18,27].

The permeate flux increased when melamine and piperazine were added to the coagulation bath because they increase the hydrophilicity of the membrane surface. On the other hand, the creation of dense layers was greatly aided by the use of PEI and TiO_2_ nanoparticles in the polymeric solution production process, where the crosslinking between PEI and coating layers improves the membrane’s selective layer [35,36]. The extent of grafting of amine groups is influenced by the concentration and the molecular weight of polyethyleneimine (PEI). With constant molecular weight, salt rejection increased noticeably, whereas the water flux decreased with an increase in the PEI grafting concentration. Also, PEI increases the electropositivity of membrane surfaces by protonating the amino groups [35]. Nevertheless, the secondary polymerization reaction of PEI and acrylic acid is used to make the selective layer thicker, and the concentration of the amine groups enhances the hydrophilicity of the membrane surface, which subsequently increases the membrane permeability [37].

**Figure 10:**
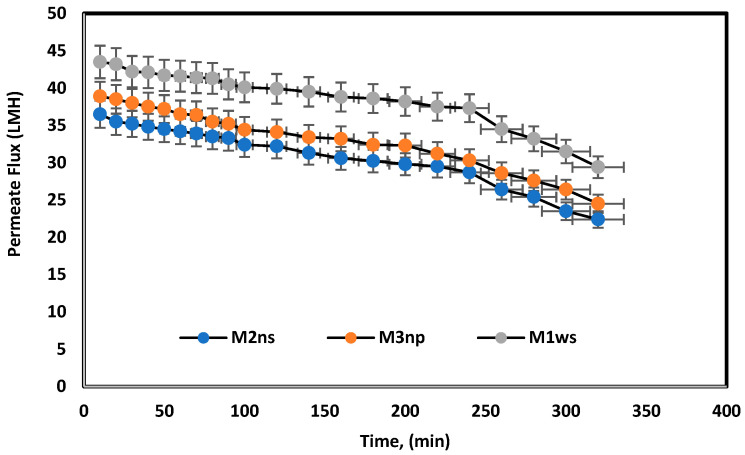
Long-term experiments for prepared membranes used melamine bath under 25 bars for 5 h.

The prepared membranes were tested using melamine as long-term experiments over a period of 5 h. Figure 10 shows a slight time dependence decrease in the permeate flux. TiO_2_ can be used to improve the antifouling properties of a membrane, as it promotes hydrophilicity, increases the self-cleaning ability of a membrane, and may raise mechanical structure capacity, when used as a filler or mechanical support [24,38]. The combination of TiO_2_ and PEI is thus proposed to increase the membrane surface antifouling based on controllable surface charge [23]. Table 3 compares the prepared membranes in the previous literature and this study, where blended cellulose acetate membranes have lower permeated flux compared to TFC membranes and polyamide membranes using nanoparticles. Nevertheless, the membranes in this article demonstrate permeate flux close to the polyamide membrane flux in woven and non-woven polyester supports.

Unsupported membranes have poor mechanical properties in comparison to support prepared membranes. The supported commercial membranes are fabricated to withstand high pressures and the limitation of desalination plants’ operation. Indicatively, the tensile strength of an unsupported polyethersulfone (PES) membrane was found to be 6.1 MPa, and the tensile strength of the supported membranes in this study was 38.53 MPa with an unwoven polyester support [33].

## 4. Conclusions

Different kinds of fabric support were used in this work to produce high-performance RO membranes. The woven polyester support shows the highest mechanical properties of 37.3 MPa and 18.5%, while the non-woven polyester provides 12.5 MPa and 17.5% for tensile strength and elongation, respectively. However, the hydrophobic nature of the support provides a good chemical bond between the support and the membrane polymeric matrix. The performance results indicate that M1ws provides the highest permeate flux for all prepared membranes on woven support, where the permeate flux reached 31.1, 39.9, and 38.2 L/m^2^.h for the prepared membranes in different water, piperazine, and melamine baths, respectively. M2ns exhibits the highest salt rejection percentage, which was 85.5, 87.6, and 92.2% for the prepared membranes in different baths: water, piperazine, and melamine baths, respectively. This work indicates a good selective membrane that was prepared on the polyester non-woven support, demonstrating gains in terms of high rejection percentage, good permeate flux, and good mechanical properties.

This paper shows that by introducing TiO_2_ and polyethyleneimine (PEI) into the polymeric membrane structure supplemented with fabric support materials, it is possible to achieve membranes with improved mechanical strength and antifouling behavior. These membranes can be used in different applications, such as desalination, the removal of heavy metals, bacterial filtration, and separation of organic compounds. The high performance of these membranes allows them to accommodate varying production conditions, maintain continuous processing in methods like tangential flow filtration, and assume greater scales of industry in terms of products and yields when compared to traditional strategies.

## Figures and Tables

**Table 1:** Prepared membranes using different supports.

Membrane Symbol	Support Type
M1ws	Woven polyester
M2ns	Non-woven polyester
M3np	Non-woven polypropylene

**Table 2:** SEM and contact angle for using fabric supports.

Symbol	SEM Images		Average Fiber Thickness (µm)	Contact Angle	Types of Fabric
Polyester	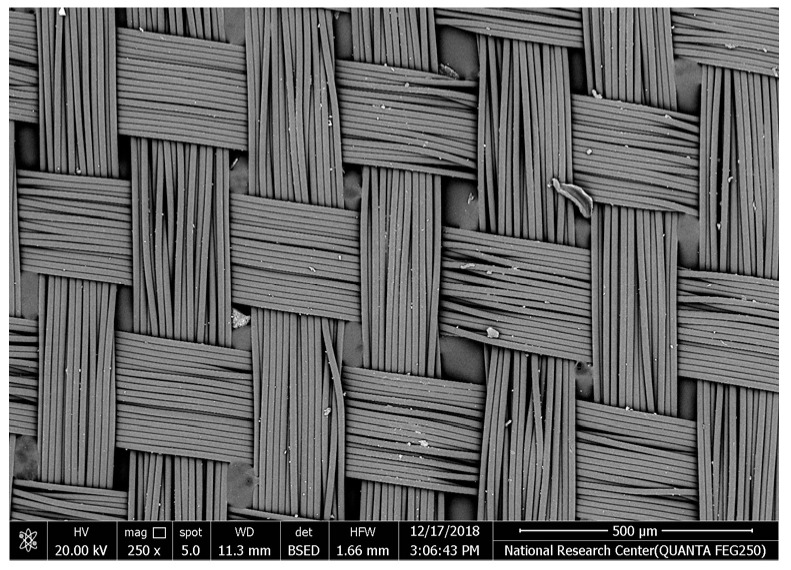	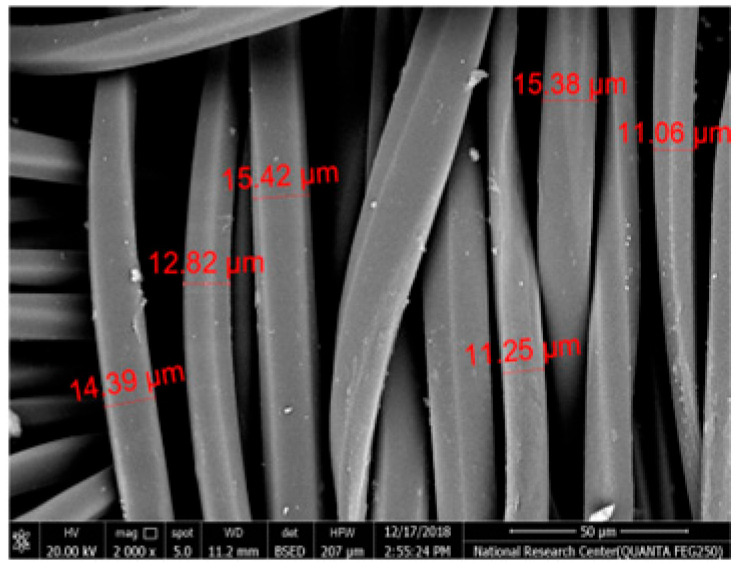	13.24	84.5°	woven
Polyester	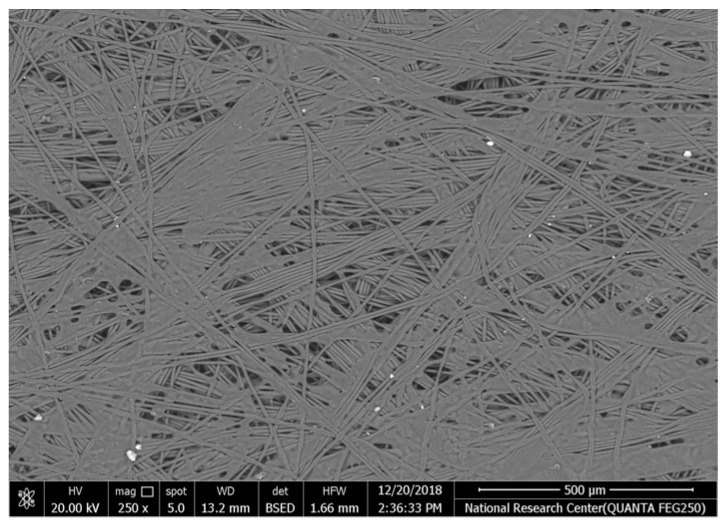	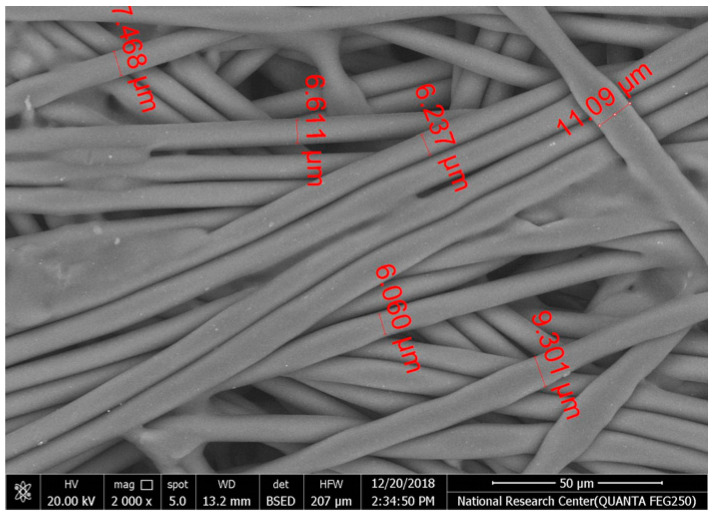	8.58	89.6°	Non-woven
Polypropylene	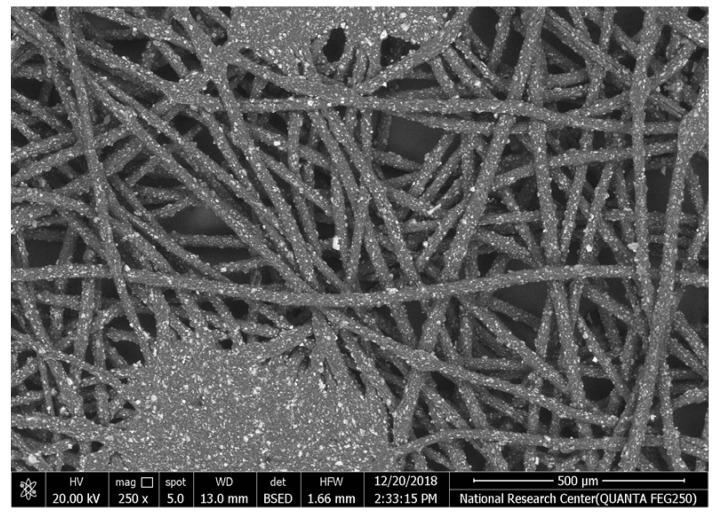	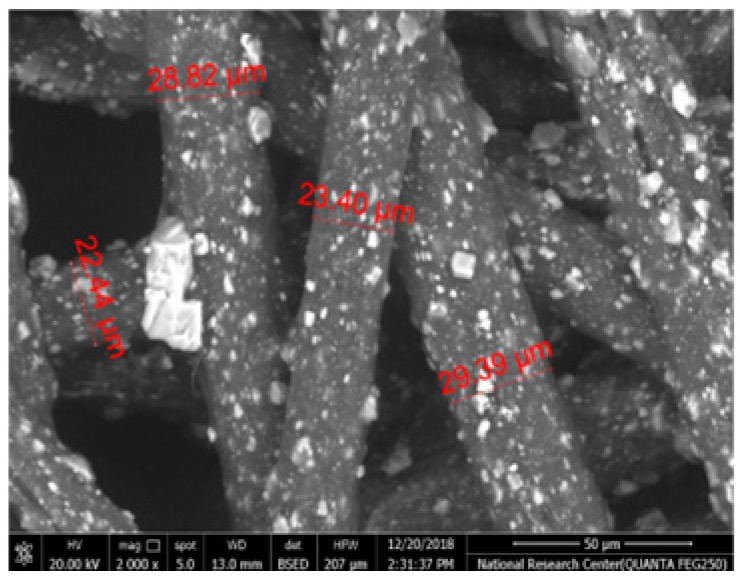	25.92	93.4	Non-woven

**Table 3:** Comparison between previous RO membranes in research and membranes in this work.

Membrane	Nanoparticle	Permeate Flux (LMH)	NaCl Salt Rejection (%)	Ref.
Cellulose acetate/polyvinyl alcohol	ZrO_2_	12.5	97	35
Polyamide membrane	ZnO	30.2	97.21	36
TFC	GO/SiO_2_	44.5	81.44	37
Polyamide membrane	CNT	37.2	95.4	38
Cellulose acetate polyvinylidene fluoride	UiO-66	5.7	90.7	39
Layer by Layer PES	TiO_2_/PEI	34.5	92.2 (Non-woven support)	This work
Layer by Layer PES	TiO_2_/PEI	40	86.6 (woven support)	This work

## Data Availability

The original contributions presented in this study are included in the article. Further inquiries can be directed to the corresponding author.
